# Processes of hypoallergenic crops at agricultural biotechnology

**DOI:** 10.1186/2045-7022-1-S1-P38

**Published:** 2011-08-12

**Authors:** Sebnem Kavakli

**Affiliations:** 1 Ege University Graduate School of Natural and Applied Sciences, Biotechnology, Izmir, Turkey

## 

Food allergies are a major health concern in industrialized countries.Reduction of allergens in foods, either by food processing or genetic engineering are strategies to minimize the risk of adverse reactions for food allergic patients.Biotechnological approaches can use for the reduction of allergens in plant foods. Because food allergens can be life threathening, a variety of strategies to abrogate or minimize allergic episodes are currently under study. Hypoallergic crops can use at food allergies.On the other hand, no hypoallergenic crops are commercially available.

Some crops, such as rice, tomato, apple and in legume ones, several allergens have been targeted fo reduction, including Lyc e 1, Lyc e 3, Mal d 1, cysteine protease, Ara h 2.

There are two different process for obtain hypoallergenic crops. In the first, germplasm lines are screened for the absence or reduced content of specific allergenic proteins.In the second, genetic transformation is used to slience nature genes encoding allergenic proteins.

Germplam screening can be applicable at protein and DNA levels. At protein level, protein screens have been performed using specific allergen or stained gels that evaluate the overall protein profile of varieties of interest.At DNA level of germplasm screening; the amplification of the gene of interest from a pool of template DNAs that could carry natural mutations, and the main objective has been to identify cultivars carrying natural hypoallergenic variants of known allergens.

Genetic transformation have been developed using RNA interferance (RNAi).It is a post transcriptional gene silencing (PTGS) technique.PTGS is induced by sence transgene that can suppress expression of the transgene as well as the endogenous homologous genes, hence the name cosuppression. This study aims to explain the processes which use for producing hypoallergic crops and to give examples of applications about the subject.

